# Prevalence and determinants of adolescent tobacco smoking in Addis Ababa, Ethiopia

**DOI:** 10.1186/1471-2458-7-176

**Published:** 2007-07-25

**Authors:** Emmanuel Rudatsikira, Abdurahman Abdo, Adamson S Muula

**Affiliations:** 1 Departments of Global Health, Epidemiology and Biostatistics, Loma Linda University School of Public Health, Loma Linda, California, USA; 2 UNICEF Country Office, Addis Ababa, Ethiopia; 3 Department of Community Health, University of Malawi, College of Medicine, Blantyre, Malawi

## Abstract

**Background:**

Tobacco smoking is a growing public health problem in the developing world. There is paucity of data on smoking and predictors of smoking among school-going adolescents in most of sub-Saharan Africa. Hence, the aim of this study is to estimate the prevalence of smoking and its associations among school-going adolescents in Addis Ababa, Ethiopia.

**Methods:**

Data from the Global Youth Tobacco Survey (GYTS) 2003 were used to determine smoking prevalence, determinants, attitudes to, and exposure to tobacco advertisements among adolescents.

**Results:**

Of the 1868 respondents, 4.5% males and 1% females reported being current smokers (p < 0.01). Having smoking friends was strongly associated with smoking after controlling for age, gender, parental smoking status, and perception of risks of smoking (OR = 33; 95% CI [11.6, 95.6]). Male gender and having one or both smoking parents were associated with smoking. Perception that smoking is harmful was negatively associated with being a smoker (odds ratio 0.3; 95% confidence interval, 0.2–0.5)

**Conclusion:**

Prevalence of smoking among adolescents in Ethiopia is lower than in many other African countries. There is however need to strengthen anti-tobacco messages especially among adolescents.

## Background

Smoking, which is the major single known cause of non-communicable diseases [1-5], is widespread around the world. The World Health Organization (WHO) estimates that about 30% of the adult male global population smokes [6]. National smoking prevalence among men in sub-Sahara Africa vary from 20% to 60% and the annual cigarette consumption rates are on the rise for both men and women [7]. Among sub-Saharan African youth, rates of smoking range from 1.4% in Zimbabwe and 1.5% in Nigeria to 34.4% in Cape Town, South Africa, which is cause for concern [8]. In Kenya, 7.2% of school-going adolescents smoke cigarettes while 8.5% use other forms of tobacco products [9]. The prevalence of smoking among young Ethiopian (15–25 years of age) living in Addis-Ababa was 11.8% for males and 1.1% for females in 1995 [10].

As the life expectancy improves in developing countries, non-communicable chronic diseases, many of which are associated with smoking, are expected to gain greater prominence. It is estimated that 50% of adolescents who start smoking become regular smokers [6]. About 50% of those who continue to smoke during adulthood die from diseases associated with smoking [11]. An estimated 250 million of today's children are expected to die from tobacco-related diseases [12].

While the Global Youth Tobacco Survey (GYTS) Collaborative Group has estimated the prevalence of tobacco smoking among teenagers in most countries [13], there is scarcity of studies on the predictors of smoking among African teenagers. In the absence of accurate data on factors associated with smoking among Ethiopian youth, the aim of this study was to estimate the prevalence of tobacco smoking and determinants of smoking among adolescents in Addis Ababa, the capital of Ethiopia.

## Methods

### Study Setting

This study was conducted in Addis Ababa, Ethiopia, a city with a population of 3.5 to 4 million. It is the official diplomatic capital of Africa with more than 90 embassies and consular representatives, which makes it the fourth diplomatic center in the world. Addis Ababa is the Headquarters of the United Nations Economic Commission for Africa (UNECA) since1988. It also houses the headquarters of Africa Union (AU), formerly the Organisation of African Unity since 1963.

### Data Collection

The data used in this study was obtained under the 2003 Global Youth Tobacco Survey (GYTS) conducted in Addis Ababa, Ethiopia. The GYTS is a school-based survey of students aged 13–15 years. It is a cross-sectional study utilizing a multistage sample design with schools selected proportional to enrollment size. Within a selected school, classrooms are chosen randomly. All the students within the selected classes are eligible for participation regardless of their actual ages. A questionnaire is self-completed anonymously by the students and this takes between 30 to 40 minutes. The GYTS uses a standardized core pertinent within their settings. The GYTS core questionnaire aims to collect the following information: prevalence of cigarette smoking and other tobacco use among young people; knowledge and attitudes of young people towards cigarette smoking; role of the media and advertising on young people's use of cigarettes; access to cigarettes; tobacco-related school curriculum; exposure to environmental tobacco smoke (ETS) and cessation of cigarette smoking. For the purpose of this study however, only data related to estimation of prevalence of smoking, associated factors and exposure to pro-tobacco advertisement are reported.

### Assessment of current smoking status

The following question was asked to assess the currents smoking status: during the past days (one month), on how many days did you smoke cigarettes?

### Ethical considerations

Permission to conduct the study was obtained from the Ministry of Education. All eligible students were also informed that participation was voluntary. Data collection was conducted in school by trained assistants without the presence of the teacher.

### Data Analysis

Data were analyzed using Stata version 9.2 (Statacorp, College Station, Texas, United States. Proportions and 95% confidence intervals were obtained as estimates of prevalence. Bivariate and multivariate logistic regression analysis was done to determine associations between current smoking status and other relevant variables according to the literature. The prevalence and mean levels were weighted to represent the total population of school going adolescents in Addis-Ababa.

## Results

### Characteristics of study participants

1868 students participated in the study of whom 1014 (56.3%) were female, and 787 (43.7%) were males. The median age was 15 years.

### Prevalence of smoking

Of the 1868 participants, 4.5% (95% CI [2.1, 3.7]) males and 1% (95% CI [0.4, 1.6]) females reported being current smokers (p < 0.01). An estimated 15.1% (95% CI ;12.6, 17.7]) and 5.7% (95% CI [4.3, 7.1]) had ever smoked a cigarette (p < 001).

Table 1 indicates that males had a more than a four-fold increase in the odds of smoking compared to females (OR = 4.6; 95% CI [2.3, 9.4]). Those with one or both parents smokers had more than two-fold increase in the odds of smoking compared to those whose parents were nonsmokers (OR = 2.7; 95% CI [1.3, 5.6]). Those with most or all friends smokers had a more than a 40-fold increase in the odds of smoking compared to those who had nonsmoking friends (OR = 42.2; 95% CI [18.8, 84.6]). Participants who perceived that smoking was harmful had 70% decrease of odds smoking (OR = 0.3 [0.2, 0.5]).

**Table 1:** Factors associated with current smoking in Addis Ababa, Ethiopia

Characteristic	Percentage of smokers	Odds ratio (95% Confidence Interval)
Age (years)		
11–12	2.9	1.00
13	1.8	0.7 [0.3, 1.7]
14	2.1	0.8 [0.3, 2.1]
15	4.7	2.0 [0.9, 4.3]
16–17	4.8	2.1 [0.9, 4.6]
Gender		
Female	1.0	1.00
Male	4.5	4.6 [2.3, 9.4]
Parental smoking status		
None	2.5	1.00
One or both parents smokers	6.9	2.7 [1.3, 5.6]
Best friend smokers		
None	1.0	1.00
Some	9.9	11.6 [5.8–23.1]
Most or all	30.6	42.2 [18.8, 94.6]
Perception that smoking is harmful		
No	8.1	1.00
Yes	2.5	0.3 [0.2, 0.5]

Table 2 indicates that participants who were exposed to tobacco adverts through billboards (51.4%), magazine (43.9%), and TV (35.3%). One in ten respondents (10.8%) reported having an item with cigarette brand on it. Compared to females, males had higher rates of those who reported having an item with cigarettes logo (p = 0.01) and exposed to tobacco adverts on billboards and magazine (0.02)

**Table 2:** Exposure to tobacco advertisements among adolescents in Addis Ababa

Characteristics	Number of participants	% of total and 95% CI
		P = 0.14
Seen cigarette brand name on TV in past 30 days	1801	35.3 (33.1–37.5)
Males	787	37.1 (33.7–40.6]
Females	1014	33.1 (30.3–36.1)
		P < 0.01
Has item with cigarette brand logo	1055	10.8 (9.16–12.8)
Males	455	13.9(11.0–17.3)
Females	600	8.6 (6.6–11.1)
		P = 0.01
Seen tobacco adverts on billboards in past 30 days	1775	51.4 (49.1–53.7)
Males	770	55.6 (51.2–60.0)
Females	1005	49.7 (46.6–52.8)
		P = 0.02
Seen tobacco adverts in newspapers/magazines in past 30 days	1761	43.9 (41.6–46.3)
Males	768	47.1 (43.5–50.6)
Females	993	41.5(33.4–44.6)

Table 3 indicates that the vast majority (90.5%) of the respondents felt that smoking is harmful. 27.6% thought that male smokers had more friends while 18.9% thought so for females. There were three times more respondents who thought that male smokers had many friends compared to those who thought so for female smokers (17.6% and 5.5%). Compared to males, females had higher rates of those who thought that smoking was harmful and male smokers had more friends (p < 0.01).

**Table 3:** Attitudes towards tobacco smoking distributed by gender in Addis Ababa

**Characteristic**	**Number of participants/Total for category**	**% of total and 95% CI**
		P < 0.01
Felt that boys who smoke have more friends	1773	27.6 [25.6–29.8]
Males	777	24.3 [21.4–27.5)
Females	996	30.2 (27.4–33.1]
		P = 0.6
Felt like girls who smoke had more friends	1767	18.9 [17.1–20.8]
Males	765	18.1 [15.6–21.1]
Females	1002	19.5 [17.2–22.1]
		P = 0.07
Felt that boys who smoke are attractive	1789	17.6 [15.9–19.5]
Males	777	18.4 [15.8–21.3]
Females	1012	17.0 [14.8–19.5]
		P = 0.2
Felt that girls who smoke are attractive	1780	5.5 [4.5–6.6]
Males	777	5.8 [4.4–7.8]
Females	1003	5.2 [3.9–6.8]
		P < 0.01
Felt that tobacco smoking is harmful to health	1783	90.5 [89.1, 91.8]
Males	778	88.0 [85.6, 90.1]
Females	1005	92.4 [90.7, 93.9]

Table 4 indicates that having smoking friends was very strongly associated with smoking after controlling for age, gender, parental smoking status, and perception of hazards caused by smoking. For those subjects who had most or all friends smokers, we found a more than 30-fold increase in the odds of smoking compared to those who had no smoking friends (OR = 33.3; 95% [11.6, 95.6]). Those who had some smoking friends had a nine-fold increase in the odds of smoking (OR = 9.0; 95% CI [4.0, 20.3]). Males had a three-fold increase of the odds of smoking compared to females (OR = 3.6; 95% CI [1.4, 8.8]).

**Table 4:** Factors associated with current smoking in Addis Ababa, Ethiopia in Multivariate analysis

**Characteristics **	**Odds ratios (OR) [95% CI]**
Age (years)	
11–12	1.00
13	0.6 [0.2, 2.3]
14	0.6 [0.2, 2.3]
15	2.5 [0.9, 7.3]
16–17	0.8 [0.7, 4.8]
Gender	
Female	1.00
Male	3.6 [1.4, 8.8]
Parents smoking	
None	1.00
One or both parents smokers	1.0 [0.3, 3.2]
Friends smoking	
None	1.00
Some	9.0 [4.0, 20.3]
Most or all	33.3 [11.6, 95.6]
Smoking harmful	
Yes	1.00
No	1.6 [0.6, 4.2]

## Discussion

The prevalence of current smoking obtained in this study was 2.9% overall, 4.5% in males and 0.4% in females. As has been demonstrated in other studies [14-16], males had a higher prevalence of smoking than females. Our estimates are at the lower end of the range of the prevalence of smoking among sub-Saharan youth [7]. Mpabulungi and Muula have reported overall smoking prevalence of 21.9%, 12.2% in females and 25.5% in males in Arua, Uganda, but much lower prevalence in Kampala (5.3%), the capital city [17,18]. It seems that Ethiopia has maintained a low prevalence of smoking among young people as had been reported in the 1980s and 1990s [10,19].

We found it notable that participants who believed that smoking was harmful to health had lower likelihood of being smokers compared to those who did not (OR= 0.3 95% CI 0.2–0.5). This probably suggests that anti-tobacco messages among young people are effective in discouraging tobacco use. It may also result from the positive influence of role models who have led those children to believe that smoking is harmful. As having a parent who is a smoker was associated with being a current smoker, this suggests the influence that parents have on their children lifestyles. The fact that current smoking was also associated with best friend being a smoker could either suggest peer influence in initiating smoking or that smokers are likely to be-friend other smokers. Either way however, it is likely that having a smoking friend is a major marker of being a smoker oneself.

Despite the relatively lower prevalence of smoking in Addis Ababa compared to other settings in Africa, adolescents are increasingly being exposed to pro-tobacco advertisements in the media, billboards and other means as shown in Table 2. Glorification of smoking in films has potential to influence smoking initiation among youth [20].

Between 17% and 28% of participants felt that boys who smoke had more friends or were attractive. The perceived positive image that smokers may have could influence initiation and maintenance of smoking among adolescents. Clark et al, have reported on participants' concerns with weight gain in a smoking cessation program. [21]. Concerns about body image are important considerations among young people who perceive that smoking enhances their image. There is therefore need to appraise young people with knowledge about the short and long-term harmful effects of smoking.

The reasons behind the relatively lower prevalence of smoking among adolescents in Ethiopia are not clear. In Uganda, the differences between smoking prevalence in rural Arua and urban Kampala have been in part explained by the fact that Arua is a large tobacco growing area where smoking permissiveness is higher. Davies has reported on the dependency of the economy on tobacco income in Malawi and the consequent difficult to promote anti-smoking efforts [22]. In Ethiopia in 1977 commercial production tobacco accounted for 5% of the total industrial gross value of production and over 1% of the total number of employees in industry and accounting for 1.6% of total government revenue [19]. Tobacco continues to be a major industry in Ethiopia. However, this is not so much a factor within Addis Ababa.

Our study has several limitations. Firstly, the GYTS relies on self-completion of the questionnaires. The accuracy of reporting in this study is not known. However, Brener et al has reported high reliability of results on teenage smoking when questionnaires are administered and self-completed [23]. In our study, no biomarkers such as cotinine levels or exhaled carbon monoxide were done to validate exposure to tobacco either through self use or environmental exposure [24-28].

All study participants were recruited from schools. Interpretation of the results to the general adolescent population in Addis Ababa must be made with caution as school-going adolescents may not be representation of the overall adolescent population. The gross enrollment ratio (GER) in primary and secondary schools in Ethiopia is estimated at between 16% to 28% [29]. The GER is the number of children enrolled in a level (primary or secondary), regardless of age, divided by the population of the age group that officially corresponds to the same level. The gross enrolment ratio (GER) can be higher when there are high levels of repetitions though. The fact that school enrolment for age is low suggests that a large proportion of adolescents in Ethiopia do not attend school. It is possible that our sample may have a different smoking prevalence than those not attending school.

The nature of the GYTS is that only students present on the day of the survey are interviewed, thus excluding those eligible but absent on the day of the survey. If smokers are likely to be absent, then our prevalence under-estimates the actual level of smoking. On the other hand if non-smokers are likely to be absent, then our estimates would over-estimate actual smoking levels. It is however more likely that smokers would skip school and so our estimates are likely to be conservative.

Many tobacco cessation initiatives in Addis Ababa are adult-oriented. Foraker et al however, have reported that perception of lack of appropriate programs hinder cessation of smoking. Cultural practices may also prevent access to care [30]. There is need to provide age and gender-specific smoking cessation programs for adolescents in Ethiopia.

## Conclusion

The prevalence of smoking in Addis Ababa, Ethiopia is much lower than other setting in Africa. There is however need to reduce the current levels. Identification of factors why smoking has been maintained at such low levels in Addis Ababa could guide anti-tobacco initiatives in other parts of Africa.

## Competing interests

The author(s) declare that they have no competing interests.

## Authors' contributions

ER participated in data analysis, writing and revising the manuscript.

AA was responsible for data collection, initial analysis and drafting of manuscript.

ASM was responsible for conception of the present data analysis, and participated in writing manuscript.

Although AA is currently affiliated with UNICEF, the organization had nothing to do with the work, nor decision to publish.

All authors reviewed and approved manuscript.

## Pre-publication history

The pre-publication history for this paper can be accessed here:


